# Effect of a multichannel oral irrigator on periodontal health and the oral microbiome

**DOI:** 10.1038/s41598-023-38894-0

**Published:** 2023-07-25

**Authors:** Jin Man Kim, Soo-Yeon Yoo, Jung-Sub An, Jihee Jessica Woo, Young-Dan Cho, Hwi Eun Park, Myong-Hwan Karm

**Affiliations:** 1 Department of Oral Microbiology and Immunology, School of Dentistry and Dental Research Institute, Seoul National University, 101 Daehak-ro, Jongno-gu, Seoul, 03080 Republic of Korea; 2 Department of Prosthodontics, Seoul National University Dental Hospital, School of Dentistry and Dental Research Institute, Seoul National University, 101 Daehak-ro, Jongno-gu, Seoul, 03080 Republic of Korea; 3 Department of Orthodontics, School of Dentistry and Dental Research Institute, Seoul National University, 101 Daehak-ro, Jongno-gu, Seoul, 03080 Republic of Korea; 4 Department of Oral and Maxillofacial Surgery and Department of Periodontics, Columbia University College of Dental Medicine, 630 West 168Th Street, New York, 10032 USA; 5 Department of Periodontology, School of Dentistry and Dental Research Institute, Seoul National University and Seoul National University Dental Hospital, 101 Daehak-ro, Jongno-gu, Seoul, 03080 Republic of Korea; 6 Department of Dental Anesthesiology, Seoul National University Dental Hospital, 101 Daehak-ro, Jongno-gu, Seoul, 03080 Republic of Korea; 7 Department of Dental Anesthesiology, School of Dentistry and Dental Research Institute, Seoul National University, 101 Daehak-ro, Jongno-gu, Seoul, 03080 Republic of Korea

**Keywords:** Microbiology, Health care

## Abstract

Oral biofilms or dental plaques are one of the major etiological factors for diverse oral diseases. We aimed to evaluate the effect of a multichannel oral irrigator (MCOI) on periodontal health in 29 participants randomly divided into two groups: the MCOI group and the control group. To evaluate the effect of the MCOI on periodontal health, the modified Quigley Hein Plaque Index (PI), Mühlemann-Son Sulcus Bleeding Index (SBI), bleeding on probing (BOP), and swelling were evaluated and compared before and after MCOI use for 3 days. Although PI and SBI showed statistically significant increases in the control group, the MCOI group showed no significant changes in either parameter. Moreover, the percentage of BOP was significantly lower in the MCOI group. Saliva samples were analyzed by 16s rRNA amplicon sequencing to investigate changes in the oral microbiome. Sequencing results showed that *Porphyromonas* spp. were significantly increased in the control group, whereas no significant change was detected in the MCOI group. Using the MCOI, enriched populations and functional pathways were detected in pioneer species comprising non-mutans streptococci. These findings provide evidence of the effectiveness of the MCOI in maintaining periodontal health and a healthy microbial ecology in the oral cavity.

## Introduction

Oral biofilms or dental plaques are one of the major etiological factors for diverse oral diseases, such as gingival inflammation and dental caries. Formation of the biofilm begins 1 h after oral rinsing. Then it is assumed to mature within approximately 72 h, and the dynamics and composition could be affected by the diet and immunity of the host^1^. As the complexity and volume of the biofilm increase, combined with host susceptibility, oral pathogenesis may occur. The recurrent inflammatory state in the gingiva allows an uncontrolled immune reaction of the host, and the immune response against microbial dysbiosis induces collateral damage to supporting structures around the teeth, resulting in periodontitis^2^. Previous studies have demonstrated that periodontal disease is a consequence of chronic bacterial infections^3,4^. Oral biofilms in healthy conditions are mainly comprised of gram-positive facultative anaerobes such as *Streptococcus* spp.; however, in pathologic conditions, the population of microbiota is switched to gram-negative obligate anaerobes (e.g., *Porphyromonas*, *Treponema*, and *Tannerella* spp., called red complex^5^)*.* Concerning the relationship between oral biofilm and dental caries, previous studies indicated that increased caries risk is associated with an altered population in the biofilm with a high capacity for acidogenesis and acid tolerance. Such bacteria include lactobacilli, mutans streptococci, and other types of acidic organisms. The dysbiosis of oral microbiota in the biofilm can be a key event in opportunistic infection, resulting in periodontal destruction beginning with gingival inflammation, characterized by color change, swelling, and bleeding of the gingiva^6,7^ as well as dental caries^8^. Therefore, biofilm removal is the most critical approach for maintaining oral health and can be readily addressed using mechanical toothbrushing^9^.

By using conventional toothbrushing with cleansing agents, pathogens in the biofilm can be significantly reduced by disrupting the integrity of the pathological organism network while maintaining commensal bacteria. Mechanical toothbrushing in any form is considered the gold standard for removing dental plaque^10,11^. However, toothbrushing only removes 42% to 60% of the total biofilm in the oral cavity because it can be difficult for even average people to perform this effectively and thoroughly^10^. Proper toothbrushing instructions and supplementary oral care products are needed to improve the oral hygiene of each individual^12,13^. Products for oral irrigation, such as one-channel oral irrigators, involve the direct application of a pulsating stream of water to remove the oral biofilm in hard-to-reach areas in the mouth^14^. The oral irrigation system has been demonstrated to reduce the probing pocket depth, bleeding on probing (BOP), supragingival plaque, and pro-inflammatory cytokine levels^15^. Oral irrigators are designed to aid in the cleaning of the oral cavity and have been shown to be safe and effective tools for improving and maintaining oral health^14–17^.

The disadvantages of these devices are that they must be placed at the exact location where irrigation is required, and they have the risk of aspiration due to the lack of suction function. Aspiration pneumonia is fatal in disabled and elderly people^18,19^. Recently, a multichannel oral irrigator (MCOI) (COMORAL®, SMDsolutions Co., Ltd., Seoul, Korea) designed for gingival crevice cleaning was introduced. Compared to previous irrigators with a single nozzle, the MCOI has dozens of injecting holes in the mouthpiece through which injected water can flow simultaneously. Additionally, according to the manufacturer, the MCOI provides a suction function synchronized with water ejection to prevent the aspiration of bacteria-containing water into the lungs.

This study aimed to evaluate the effectiveness of a new oral hygiene device (MCOI) on periodontal health. By performing 16s rRNA amplicon sequencing and shotgun metagenomic sequencing, changes in the oral microbiota were also investigated.

## Results

### Clinical effect of the new oral hygiene device (MCOI) on gingival indices

**Figure 1:**
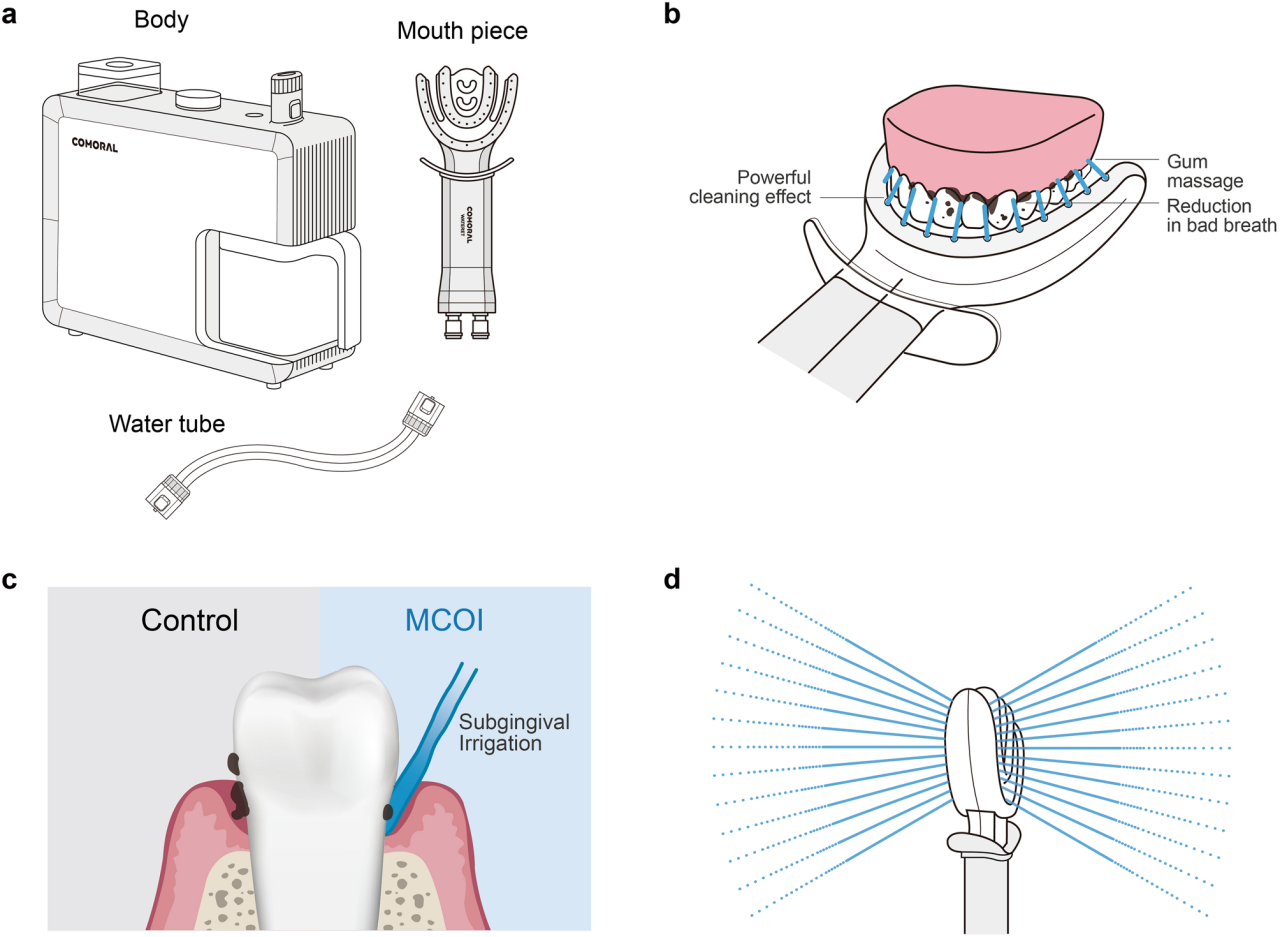
Multichannel oral irrigator (MCOI) device used in this study. (**a**) The new MCOI device for maintaining oral hygiene comprises 3 parts: body, mouth piece, and water tube. (**b**) Mouthpiece: it injects clean water onto the teeth at an angle of 45°, targeting the gingival margin and sucking the remaining water. (**c**) The MCOI aims to irrigate the subgingival remnant. (**d**) Mouthpiece head: it injects clean water onto the teeth at an angle of 45°.

**Figure 2:**
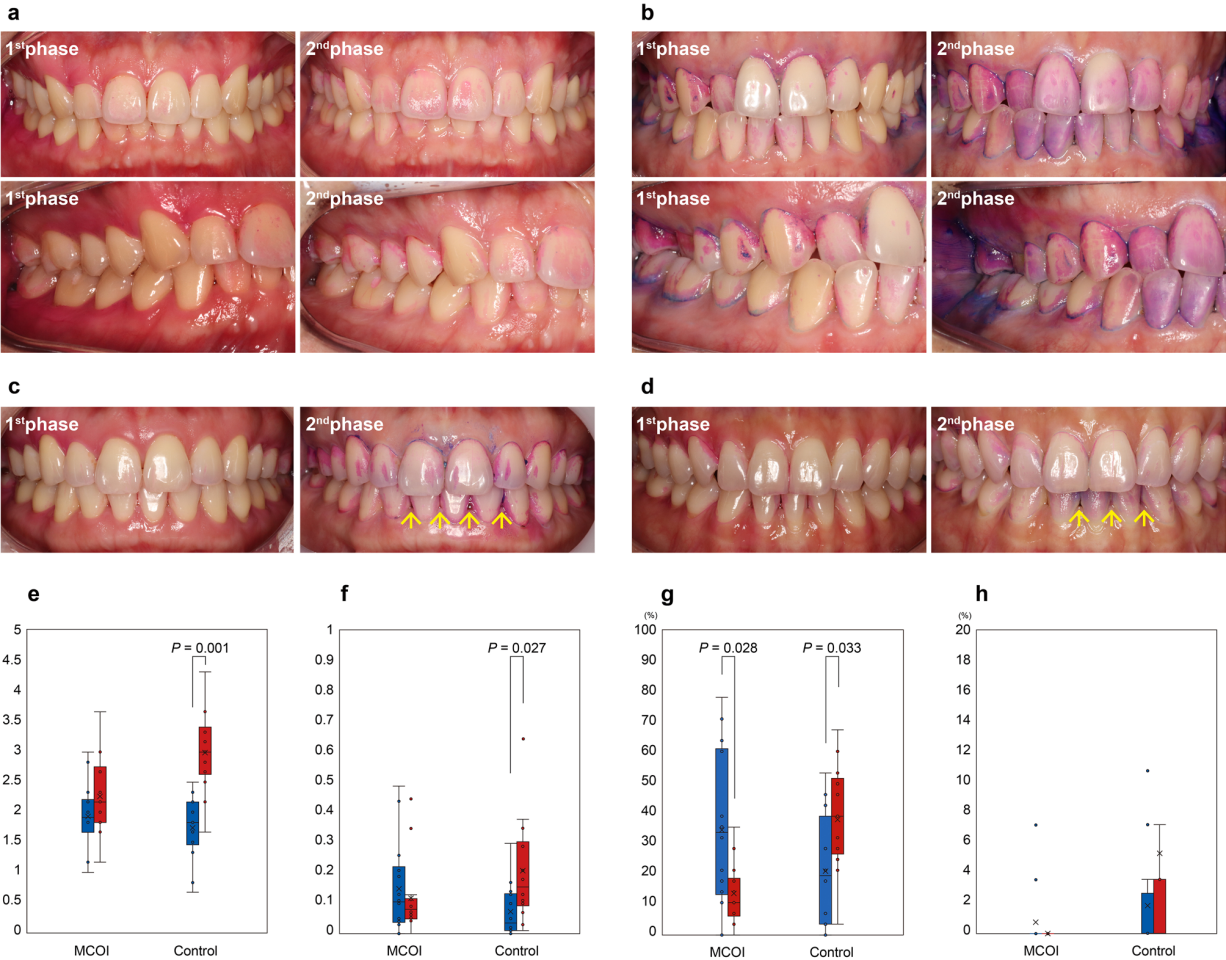
Periodontal evaluation in the clinical trial. (**a**) Representative case in the multichannel oral irrigator (MCOI) group. (**b**) Representative case in the control group. (**c** and **d**) Supra-gingival calculus (arrows) is observed in the mandibular anterior teeth after 3 days in only the control group. (**e**) Modified Quigley Hein Plaque Index, range 0–5. (**f**) Mühlemann-Son Sulcus Bleeding Index, range 0–5. (**g**) Percentage of bleeding on probing (BOP) in all teeth. (**h**) Percentage of swelling in all teeth. The blue bars indicate values in the preclinical trial, and the orange bars represent values in the postclinical trial.

Twenty-nine of 30 participants were finally included for clinical measurements; 1 participant was excluded because of loss during the analysis process. The mean age of participants in this clinical examination was 33.7 ± 8.2 years. Finally, 16 women and 13 men were included in this clinical trial. Table 1 shows the demographic data of the study population. The study protocol and measurements are shown in Supplementary Fig. S1. Figure 1 shows the MCOI used in this study. Figure 2 shows representative cases from the MCOI and control groups. Supra-gingival calculus was observed in only the control group after 3 days of the clinical trial (Fig. 2a–d). Table 2 shows the changes in gingival status. In the control group, the average PI and SBI significantly increased (Fig. 2e,f). In the MCOI group, the percentage of BOP significantly decreased (Fig. 2g), and that of swelling also decreased; however, no significant difference was noted (Fig. 2h). Four mandibular anterior teeth (central and lateral incisors) in 2 of 14 participants in the control group showed mild supra-gingival calculus extending to the marginal gingiva (Fig. 2c,d).

**Table 1:** Demographic data of study population.

	Total (N = 29)	MCOI (N = 14)	Control (N = 15)	P-value
Age (years)	33.7 ± 8.2	35.5 ± 10.4	32.0 ± 5.2	0.271
Sex				1.000
Female	16 (55.2)	8 (57.1)	8 (53.3)	
Male	13 (44.8)	6 (42.9)	7 (46.7)	
Height	168.1 ± 7.2	166.2 ± 8.1	169.9 ± 6.1	0.171
Weight	64.8 ± 12.8	65.1 ± 13.7	64.5 ± 12.3	0.901
Smoking	0	0	0	
Alcohol consumption*	13 (44.8)	6 (42.9)	7 (46.7)	1.000
Sweet snack^†^	27 (93.1)	13 (92.9)	14 (93.3)	1.000
Sleep disturbances^‡^	4 (13.8)	3 (21.4)	1 (6.7)	0.540
Respiratory disease	0	0	0	

Values are expressed as the mean ± standard deviation or number (percentage). MCOI, multichannel oral irrigator. *This variable checked social drinking, and most of the respondents consumed one or two bottles of beer once or twice a week. ^†^This variable checked whether they ate sweet snacks in addition to meals, and the respondents answered once or twice. ^‡^These variables were sleep apnea in two, bruxism in one, and snoring in the other.

**Table 2:** Changes in gingival status.

	MCOI (N = 14)		*P*-value	Control (N = 15)		*P*-value
	Pre	Post		Pre	Post	
PI	1.92 ± 0.57	2.26 ± 0.63	0.151	1.67 ± 0.56	2.91 ± 0.70	0.001
SBI	0.14 ± 0.16	0.11 ± 0.10	0.972	0.07 ± 0.08	0.20 ± 0.17	0.027
BOP	34.69 ± 25.92	13.77 ± 9.89	0.028	18.87 ± 18.44	33.80 ± 20.06	0.033
Swelling	0.76 ± 2.06	0	0.180	1.67 ± 3.35	4.52 ± 12.22	0.359

Values are expressed as the mean ± standard deviation or number (percentage). BOP, bleeding on probing; MCOI, multichannel oral irrigator. PI, Quigley Hein Plaque Index; SBI, Mühlemann-Son Sulcus Bleeding Index.

### Effect of the MCOI on oral microbiota

**Figure 3:**
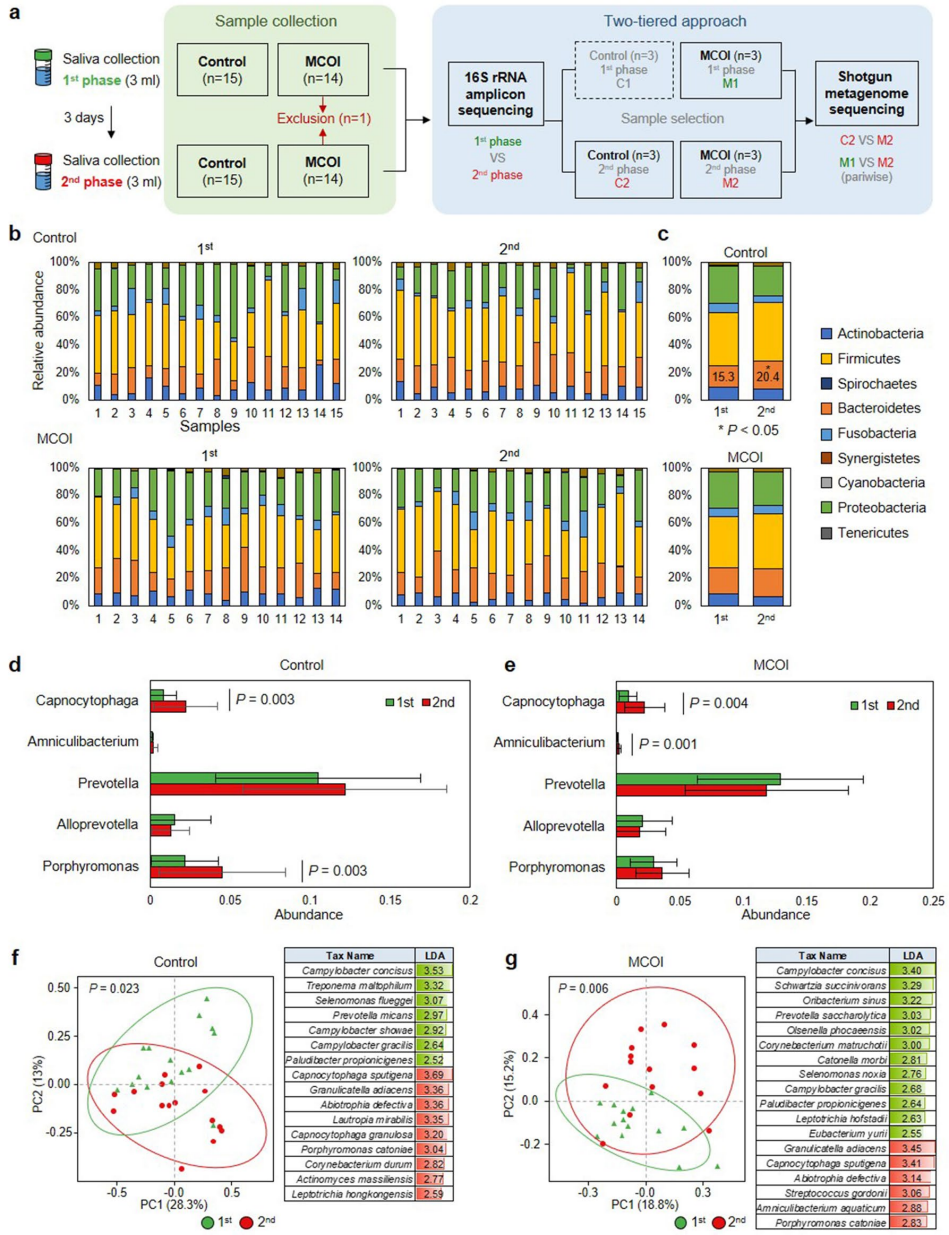
Results of 16s rRNA amplicon sequencing of the control (no brushing) and multichannel oral irrigator (MCOI) groups. (**a**) Study design of sample collection and the two-tiered approach combining 16s rRNA and metagenomic sequencing. (**b**) Relative abundance of oral microbiota in individual samples of the control and MCOI groups at the phyla level. (**c**) Average relative abundance of oral microbiota in the control and MCOI groups at the phyla level. n = 15 (control) and 14 (MCOI), *P < 0.05. (**d** and **e**) Average relative abundance of 5 selected genera in the first and second phases in the control (**d**) and MCOI (**e**) groups. n = 15 (control) and 14 (MCOI); data are presented as mean ± standard error of the mean. (**f** and **g**) Principal coordinates analysis (left) and linear discriminant analysis effect size analysis (right) of the control (**f**) and MCOI (**g**) groups. *P*-values were calculated by analysis of similarities.

Given the clinical results, we designed an experimental process based on a two-tiered approach^20^ to dissect the salivary microbiome. In detail, collected saliva samples were analyzed by 16s rRNA amplicon sequencing to compare the control and MCOI groups. The selected prescreened samples were analyzed in-depth by shotgun metagenome sequencing for pairwise comparison between the first and second phases in each sample (Fig. 3a). First, the relative abundance of amplicon sequence variants (ASVs) in saliva samples was profiled at the phylum level by 16s rRNA amplicon sequencing (Fig. 3b and see Supplementary Fig. S2a online). There was no significant difference in the alpha diversity between the groups (see Supplementary Fig. S2b online). By comparing the average distribution, a significant increase in the phylum *Bacteroidetes* (15.3 ± 6.98% versus [vs.] 20.4 ± 5.19%; *P* = 0.031) was observed in the control group, whereas the MCOI group maintained a distribution similar to that of the basal state (Fig. 3c). The phylum *Bacteroidetes* includes multiple pathogenic species in the human oral cavity, such as *Prevotella* and *Porphyromonas*^5,21^. Therefore, we investigated changes in the *Bacteroidetes* subpopulation at the genus level based on the sequencing database. *Captocytopaga* spp., which are opportunistic pathogens involved in periodontal disease^22^, were significantly increased in both groups (Fig. 3d). Notably, *Porphyromonas* spp. showed a significantly increased abundance by 114.28% (*P* = 0.003) in the control group, indicating the possibility of disease progression in the periodontium (Fig. 3d). However, no significant change in the distribution of *Porphyromonas* was detected in the MCOI group (Fig. 3e).

Next, we investigated the beta diversity between the first and second phases. The significant difference between the control and MCOI groups was demonstrated by principal coordinate analysis with analysis of similarities, with *P*-values of 0.023 and 0.006, respectively (Fig. 3f,g). Differences in the distribution of bacterial species were measured using a linear discriminant analysis (LDA) score (see Supplementary Fig. S3a–d online). The results showed that *Streptococcus gordonii*, a representative pioneer species on the tooth surface belonging to oral commensal bacteria, was significantly increased in the MCOI group (Fig. 3g; LDA score = 3.06). Together with *S. Gordonii*, *S. Oralis* presented a clearly increasing pattern, but with no significance (see Supplementary Fig. S3e online; *P* = 0.058). In the control group, pioneer streptococci showed high individual variance with no significance between the first and second phases (data not shown). Cariogenic spp. belonging to mutans streptococci (*S. mutans* and *S. sobrinus*) were seldom detected in either group. Collectively, the 16s rRNA sequencing results revealed the relevant changes in oral microbiota during the first and second phases, even with a short observation period; the oral microbiota was altered to a more favorable population for periodontal disease without toothbrushing, whereas recycling patterns in oral commensal bacteria were observed after MCOI treatment.

### Effect of the MCOI on the oral microbiome

**Figure 4:**
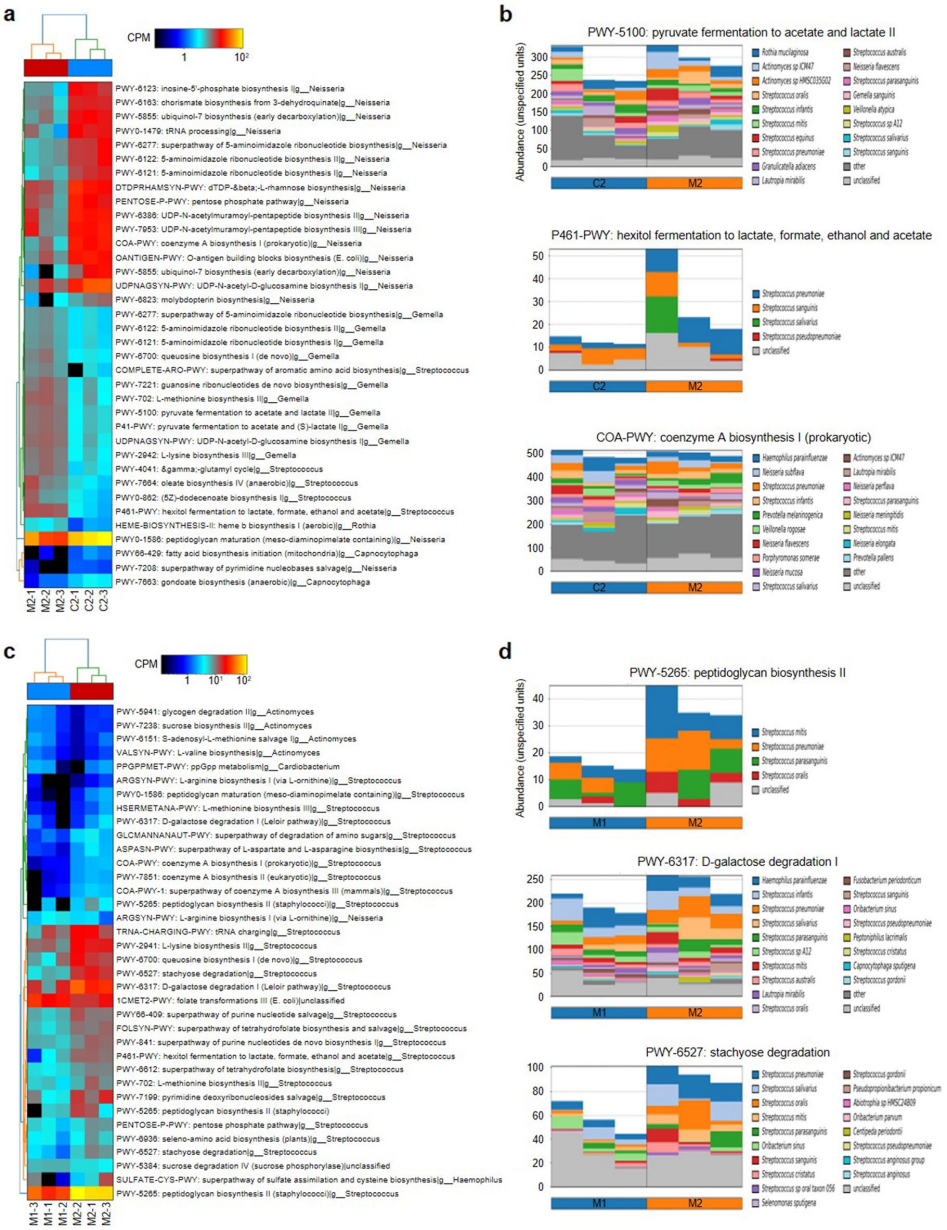
Shotgun metagenomic sequencing of the representative samples. (**a**) Heatmap of functional pathway analysis comparing the control and multichannel oral irrigator (MCOI) groups in the second phase. Full lists are provided in the supplementary data. CPM, counts per million mapped reads. (**b**) Pathway bar plots of the control and MCOI group in the second phase. Stacked bar plots illustrate the relative abundance and contribution diversity in the pathways across the samples. (**c**) Heatmap of functional pathway analysis comparing the first and second phases in the MCOI group. (**d**) Pathway bar plots of the first and second phases in the MCOI group.

To further investigate the functional aspects of changes in the microbiota, we performed shotgun metagenomic sequencing using selected representative samples from the control and MCOI groups (Fig. 3a, see Materials and Methods). We first compared the control and MCOI samples in the second phase (C2 vs. M2, Fig. 3a) by metabolic pathway analysis. The MCOI group showed an increased abundance of various metabolic pathways related to amino acid synthesis, energy metabolism, and nucleotide biosynthesis almost exclusively in gram-positive bacteria, such as *Streptococcus*, *Gemella*, and *Rhothia* spp. (Fig. 4a). The fermentation pathways related to acid production were enriched in *Streptococcus* and *Gemella* spp., but no species with high acidogenicity (e.g. mutans streptococci) was detected (Fig. 4a,b). Conversely, multiple biosynthetic pathways were reduced in gram-negative bacteria in the MCOI group, represented by *Neisseria* and *Capnocytophaga* spp. (Fig. 4a,b). Next, we conducted a pairwise comparison between the first and second phases in the MCOI group (M1 vs. M2, Fig. 4c). After using the MCOI, multiple functional pathways of non-mutans streptococci generally increased, as indicated by cell wall component synthesis (PWY-5265) and carbohydrate metabolism (PWY-6317 and PWY-6527) (Fig. 4d). Notably, the increased functionality in pioneer species corresponded to the 16S sequencing results (Fig. 3g). A reduced abundance of functional pathways was observed in *Actinomyces* spp. (Fig. 4c).

## Discussion

Herein, the clinical effectiveness of the MCOI in improving oral hygiene was evaluated, with a focus on gingival inflammation. First, the PI was measured to detect the change in the volume of dental plaque using this new device. In both groups, there was an increase in dental plaque after the clinical trial, although the PI of only the control group was statistically significant (Fig. 2e). After 3 days of the clinical trial, 14.28% of participants in the control group showed supragingival calculus in 4 mandibular anterior teeth (central and lateral incisors), whereas none of the participants in the MCOI group showed calculus. It is assumed that water irrigation using this new device might help suppress the accumulation of oral biofilms. However, the effectiveness of this new device in decreasing dental plaque cannot be determined because of the accumulation of plaque in the MCOI group.

The SBI showed a significant increase in gingivitis only in the control group after the clinical trial (Fig. 2f). Although the SBI has been widely used as an indicator of gingival and periodontal status, there is some disagreement among investigators regarding the representativeness and significance of this parameter^23^. It is suggested that investigators should examine the gingival inflammation components of three basic criteria (BOP, swelling, and color change) individually. BOP and swelling can be assessed fairly objectively; however, the assessment of change in gingival color is subjective and should not be used according to the recommendations of the World Health Organization^24^. Objective criteria such as supra- and subgingival calculus and pocket depth would also be good methods for epidemiological surveys of periodontal status^25^. Hence, this study focused on fundamental criteria rather than the gingivitis index and evaluation of BOP, swelling, and calculus. This preliminary study lasted three days and was focused on gingival status change. Longer-term clinical studies, on the other hand, should include critical periodontal indicators such as pocket depth and clinical attachment level (CAL).

After the clinical trial, BOP was significantly changed in both groups; in the MCOI group, BOP was decreased, and in the control group, BOP was increased (Fig. 2g). This finding indicates that gingivitis was alleviated in the MCOI group. In 2001, the American Academy of Periodontology stated, “irrigation with or without medicaments reduces gingival inflammation beyond what is normally achieved by toothbrushing alone,” which supports our results^12^. However, there was no significant change in swelling in either group. It is assumed that 3 days of a clinical trial cannot make any distinct status change in periodontal tissues.

It was determined that the attached supra-gingival biofilm on the surface of the teeth was not removed effectively; however, the subgingival biofilm was removed more efficiently by the MCOI, probably because the MCOI is designed to eject water at an angle of 45° to the gingival margin (Fig. 1c,d).

The PI and SBI can be regarded as subjective indices that can vary depending on the investigator. To address this issue, all measurements were performed randomly by a single investigator. The BOP evaluation in this study is a reliable indicator of gingival inflammation, which is useful for predicting the progression of periodontitis; however, bleeding on two or three consecutive appointments is more likely to be trusted^26,27^ than one examination conducted in this study. A 3-day clinical trial is too short to detect changes in the precise periodontal state. However, ethical reasons prevent a longer duration of a clinical trial. Gingivitis with no brushing for 3 days can be restored to normal gingiva by initiating toothbrushing again^28^. By using the MCOI twice a day for 3 days, the BOP of participants was reduced, and plaque accumulation was less than that of the control group. It is assumed that the MCOI has a positive effect on the improvement of early gingivitis. Owing to the limitation of plaque removal on the tooth surface, this new device has to be used as a supplementary tool. Additionally, further study periods and more groups, such as a toothbrushing with MCOI group, a toothbrushing only group, and a one-channel oral irrigator group, would be required for precise verification of the effectiveness of the MCOI.

Oral biofilms are the primary cause of dental caries and periodontal diseases^10,29^; they are found on the tooth surface and consist of a community of microorganisms embedded in a matrix of polymers of host and bacterial origin^30^. For a comprehensive understanding of the pathophysiology of biofilm-mediated diseases, dissecting the oral microbiome is essential, especially based on next-generation sequencing technology. In this study, we used a combined approach of 16s rRNA amplicon and shotgun metagenome sequencing, which is efficient to reveal the general profile of microbiota in the oral environment and to predict the functional aspect of the bacterial population. The two-tiered approach revealed that the use of the MCOI increases the abundance and functional pathways of pioneer species, such as *S. gordonii*, *S. oralis*, and *S. mitis*. These findings indicate the possibility of new biofilm formation due to irrigation. Moreover, metabolic pathway analysis showed reduced functionality of cariogenic bacteria, including *Neisseria* and *Actinomyces* spp., in the MCOI group. Actinomyces has been highlighted as a potential candidate for the development of proximal caries, and recently, deep metagenome sequencing indicated a high correlation between *Neisseria* and cariogenic risk^31^. *Capnocytophaga* spp., an opportunistic pathogen of periodontitis, showed dynamic changes both in abundance and functionality; taxonomic abundance was significantly increased, whereas functional pathways were generally reduced in the MCOI group. Such conflicting trends imply the need for a longitudinal study over a longer period.

The amount of biofilm in the gingival region has an immediate effect on the progression of gingivitis; therefore, removal of the biofilm is the first choice for periodontal health prevention, without concern for host susceptibility. Early intervention and removal of dental plaque or calculus are of utmost importance in preventing periodontal diseases and maintaining a healthy periodontium ^28,32,33^. However, disabled or elderly people with low dexterity have difficulty maintaining oral hygiene appropriately. Moreover, patients with Alzheimer disease, dementia, or severe autism usually lack cooperation for thorough plaque removal. The remaining bacteria in the gingival biofilm could be a considerable risk factor for periodontal disease, even in those people. Conventional toothbrushing and rinsing can cause aspiration pneumonitis due to dysphagia. Therefore, various oral care interventions might be needed to decrease aspiration pneumonia and improve oral health in the disabled and elderly populations. The recently introduced MCOI could be used for this purpose, although there was a limitation that this preliminary study only included healthy adults. It is also expected that the MCOI will expand its utility by delivering anti-inflammatory agents or antibiotics for periodontitis. Another limitation of this study is the small sample size. The study was initially planned as a randomized controlled trial involving 42 participants. However, the Institutional Review Board (IRB) recommended a reduction of the sample size because there was no preliminary study.

In conclusion, these findings provide evidence of the effectiveness of MCOI in maintaining periodontal health and a healthy microbial ecology in the oral cavity.

## Methods

### Ethics statements

All study participants provided informed consent to a pre-specified research protocol approved by IRB of Seoul National University Dental Hospital (number: CRI22005). This research was performed in accordance with the Declaration of Helsinki. All methods were performed in accordance with relevant guidelines and regulations.

### Study protocol and eligibility criteria

Thirty healthy participants were voluntarily recruited for this study. The inclusion criteria for this study were as follows: (1) those older than 19 years of age and younger than 85 years of age, without a periodontal disease or medical disease; (2) those with ≥16 remaining teeth excluding the third molars; (3) those who did not have prostheses that exceeded the range of incisal edges and occlusal surfaces such as laminates and crowns on the teeth to be evaluated; and (4) those who could voluntarily sign the consent form. Those who received local and systemic antibiotics, heavy smokers, pregnant women, persons with severe periodontal disease (> 4 mm on probing depth), those with dental caries that required restorative treatment, or persons with cognitive impairment were excluded from the study. The study protocol and measurements are shown in Supplementary Fig. S1. The recruited participants were randomly allocated to either the experimental or control group. A randomization list (www.randomization.com) was prepared by a nonparticipating member of the study.

### New MCOI device

In the experimental group, a new oral hygiene device, the MCOI, was used. This device has three compartments: a machine with pumps, a mouthpiece, and a connecting tube (Fig. 1). According to the manufacturer’s study (comoral.kr), the mouthpiece (i.e., WATERET®) of the MCOI allows water injection onto the teeth at an angle of 45°, targeting the gingival margin and delivering 500 mL of tap water at 3.71 bar of pump pressure for 1.5 min.The WATERET®’s 60 dense streams of water spray up and down in four directions, clearing foreign substances from the upper and lower teeth and within and outside the teeth. COMORAL®, the first MCOI, is categorized as a Class I medical device by the Food and Drug Administration.

### Grouping for the clinical trial

Thirty applicants who met the inclusion criteria were recruited. They were scaled and their group (MCOI vs. control group) was determined by randomization. In the first phase, 2 weeks later, all participants in the MCOI and control groups collected unstimulated whole saliva for a volume of 2–3 mL before the clinical measurements (the PI, SBI, BOP, and existence of swelling). All participants (from both groups) rinsed their mouths with one cup of tap water before the clinical examination and then held their saliva in their mouths until it flowed naturally into a 10 mL tube (Axygen Scientific, Union City, CA, US). They were told to keep spitting and collecting approximately 2–3 mL of saliva in a tube, which was immediately stored at − 80 °C.

Next, for 3 days, in the MCOI group, participants used the new oral irrigator device twice a day (in the morning and before bed) for 3 min at medium intensity. In the control group, the participants did not brush their teeth for 3 days, even after meals, before the second phase. The standard method was explained to the MCOI group participants as follows: 1. You can eat normally, with the exception of gum. 2. You should refrain from brushing your teeth. 3. Other than the MCOI provided, no other cleaning products may be used.

Participants in the control group were also strictly instructed to adhere to the following: 1. Except for gum, you can eat normally. 2. You should refrain from brushing your teeth. 3. No other cleaning products are permitted. 4. Gargle lightly with tap water three times before going to bed.

All participants were instructed as above, and in the second phase, saliva collection and clinical examinations were repeated as in the first phase.

### Clinical measurements of the PI, SBI, and BOP

All tests were conducted according to the study protocol (Supplementary Fig. S1 online). To evaluate the effectiveness of the MCOI in maintaining periodontal health, the PI^34^, the SBI^25^, BOP, and the presence of swelling were observed and compared before and after the use of the device for 3 days. At the initial visit, 30 participants were scaled by two hygienists. Then 2 weeks later, in the first phase, the PI and BOP evaluations with saliva collection were conducted for all participants. After collecting saliva from the participants, the disclosing solution (Trace® Solution, Young, MO, USA) was applied to all participants’ teeth (except the third molars) for 1 min. The PI evaluation was performed for six representative teeth (the four first molars, maxillary right central incisor, and mandibular left central incisor)^34^. The scores for the six teeth were summed, and the final score was calculated by dividing it by the number of teeth examined. To determine gingivitis, the SBI, BOP, swelling, and calculus existence were evaluated. The periodontal evaluation was conducted on all participants randomly by a single examiner to reduce errors.

### 16s rRNA amplicon sequencing

16s rRNA amplicon sequencing was performed using saliva collected from 29 participants. DNA was extracted using the DNeasy PowerSoil Kit (Qiagen, Hilden, Germany) according to the manufacturer’s instructions. The extracted DNA was quantified using a Quant-IT PicoGreen (Invitrogen, Waltham, MA, USA). Sequencing libraries were prepared according to Illumina 16S Metagenomic Sequencing Library protocols to amplify the V3 and V4 regions. The input 2 ng gDNA was amplified by polymerase chain reaction (PCR) with 5× reaction buffer, 1 mM dNTP mix, 500 nM of each of the universal F/R PCR primers, and Herculase II fusion DNA polymerase (Agilent Technologies, Santa Clara, CA). The universal primer pair with Illumina adapter overhang sequences used for the first amplification was as follows:

V3-F: 5′-TCGTCGGCAGCGTCAGATGTGTATAAGAGACAGCCTACGGGNGGCWGCAG-3′.

V4-R: 5′-GTCTCGTGGGCTCGGAGATGTGTATAAGAGACAGGACTACHVGGGTATCTAATCC-3′.

The first PCR product was purified using AMPure beads (Agencourt Bioscience, Beverly, MA, USA). Following purification, 2 μL of the PCR product was amplified for final library construction containing the index using the NexteraXT Indexed Primer (Illumina, San Diego, CA, USA). The PCR products were purified using AMPure beads. The final purified product was quantified according to the quantitative real-time (q)-PCR Quantification Protocol Guide (Illumina sequencing platforms) using the TapeStation D1000 ScreenTape (Agilent Technologies). Paired-end (2 × 300 bp) sequencing was performed using the MiSeq platform (Illumina).

Sequenced datasets were classified by index sequencing and produced in a paired-end FASTQ format. Adapter and primer sequences were removed using Cutadapt (version 3.2) (https://doi.org/10.14806/ej.17.1.200), and error-correction was conducted using the DADA2 (version 1.18.0) package^35^. The established ASVs were assigned to taxonomic information with the highest similarity based on the reference database (DB) (National Center for Biotechnology Information (NCBI) 16S Microbial DB). To examine alpha and beta diversity between the groups, the processed datasets were analyzed using quantitative insights into microbial ecology (QIIME) (version 1.9)^36^. The Wilcoxon signed rank and Kruskal–Wallis tests were also conducted based on relative abundance at the species level. Comparative analysis of relative abundance between the groups was conducted using linear discriminant effect size analysis^37^.

### Shotgun metagenome analysis

Three representative samples were selected from the C2, M1, and M3 groups based on the criteria that the samples showed the highest differences in the unweighted pair group method with an arithmetic mean tree comparing samples of 16S rRNA sequencing data (data not shown)). Sequencing libraries were prepared according to the manufacturer’s instructions using the TruSeq Nano DNA High-Throughput Library Prep Kit (Illumina). Briefly, 100 ng of genomic DNA was sheared using adaptive focused acoustic technology (Covaris, Woburn, MA, USA), and the fragmented DNA was end-repaired to create 5’-phosphorylated, blunt-ended dsDNA molecules. Following end-repair, the DNA was size-selected using a bead-based method. These DNA fragments went through the addition of a single ‘A’ base and ligation of the TruSeq DNA UD Indexing adapters. The products were purified and enriched by PCR to create the final DNA library. The libraries were quantified using qPCR according to the qPCR Quantification Protocol Guide (KAPA Library Quantification kits for Illumina Sequencing platforms) and qualified using the TapeStation D1000 ScreenTape (Agilent Technologies). We then sequenced the data using NovaSeq (Illumina).

After sequencing was completed, the raw data were demultiplexed using index sequences, and paired-end FASTQ files were generated for each sample. Adapter sequences and data with an average phred quality score < 20 were removed using trimmomatic (version 0.39)^38^ of the Kneaddata (version 0.10.0)^39^ pipeline (option: SLIDINGWINDOW:4:20). Then, to remove the human genome sequence, mapped reads to the hg37dec_v0.1 reference (human reference genome) using Bowtie2 (version 2.4.5)^40^ were removed. The preprocessed data were analyzed using MetaPhlAn4 (version 4.0.0) for approximately 1 million microorganisms composed of NCBI reference genomes and species-level genome bins. The reads were mapped to the specific marker genes of the microbial species using Bowtie2, and species abundance was calculated based on the average number of reads mapped to the marker genes. Metabolic pathway analysis was performed using HUMAnN3 (version 3.5)^41^, and the functional gene abundance of the species constructed in MetaPhlAn4 was measured based on the Pangenome database ChocoPhlAn. Reads that did not map to the ChocoPhlAn database were mapped to the UniRef90 Database using Diamond BLASTX, quantified based on the UniRef90 ID, and pathway abundance was calculated by integrating the detected functional genes.

### Statistical and data analyses

To compare the periodontal measurements (PI and SBI) before and after the clinical trial, the Wilcoxon signed-rank test was used. In addition, the equality and dominance of salivary bacteria in both groups, as well as the changes in the percentage of BOP, swelling, and calculus results, were analyzed. Statistical analyses were performed using SPSS (version 25.0; IBM Corp, Armonk, NY, USA). The significance level was set at $\mathrm{\alpha }$= 0.05.

### Supplementary Information

Supplementary Figure S1.

Supplementary Figure S2.

Supplementary Figure S3.

Supplementary Figure Legends.

## Data Availability

The datasets generated and analyzed during the current study are available in the Sequence Read Archive (SRA) repository, PRJNA937458.

## References

[1] Seneviratne CJ, Zhang CF, Samaranayake LP (2011). Dental plaque biofilm in oral health and disease. Chin. J. Dent. Res..

[2] Hajishengallis G (2014). Immunomicrobial pathogenesis of periodontitis: Keystones, pathobionts, and host response. Trends Immunol..

[3] Noiri Y, Li L, Ebisu S (2001). The localization of periodontal-disease-associated bacteria in human periodontal pockets. J. Dent. Res..

[4] Ismail FB (2015). Identification of subgingival periodontal pathogens and association with the severity of periodontitis in patients with chronic kidney diseases: A cross-sectional study. BioMed. Res. Int..

[5] Socransky SS, Haffajee AD, Cugini MA, Smith C, Kent RL (1998). Microbial complexes in subgingival plaque. J. Clin. Periodontol..

[6] Yaacob, M. *et al.* Powered versus manual toothbrushing for oral health. *Cochrane Database Syst. Rev.* 2014, CD002281 (2014). 10.1002/14651858.CD002281.pub3 PMC7133541 24934383

[7] Schmidt JC, Zaugg C, Weiger R, Walter C (2013). Brushing without brushing?—A review of the efficacy of powered toothbrushes in noncontact biofilm removal. Clin. Oral Investig..

[8] Marsh PD (1999). Microbiologic aspects of dental plaque and dental caries. Dent. Clin. North Am..

[9] Nagy P, Kövér K, Gera I, Horváth A (2016). Evaluation of the efficacy of powered and manual toothbrushes in preventing oral diseases (Systematic review with meta-analysis). Fogorv. Sz..

[10] De la Rosa M, Zacarias Guerra J, Johnston DA, Radike AW (1979). Plaque growth and removal with daily toothbrushing. J. Periodontol..

[11] Slot DE, Wiggelinkhuizen L, Rosema NA, Van der Weijden GA (2012). The efficacy of manual toothbrushes following a brushing exercise: A systematic review. Int. J. Dent. Hyg..

[12] Research, Science and Therapy Committee of the American Academy of Periodontology. T. C. o. t. A. A. o. Treatment of plaque-induced gingivitis, chronic periodontitis, and other clinical conditions. *J. Periodontol.* **72**, 1790–1800 (2001). 10.1902/jop.2001.72.12.1790 11811516

[13] Rosema NA (2011). The effect of different interdental cleaning devices on gingival bleeding. J. Int. Acad. Periodontol..

[14] Greenstein, G. & Research, Science and Therapy Committee of the American Academy of Periodontology. Position paper: The role of supra-and subgingival irrigation in the treatment of periodontal diseases. *J. Periodontol.* **76**, 2015–2027(2005). 10.1902/jop.2005.76.11.2015 16274324

[15] Cutler CW (2000). Clinical benefits of oral irrigation for periodontitis are related to reduction of pro-inflammatory cytokine levels and plaque. J. Clin. Periodontol..

[16] Sharma NC, Lyle DM, Qaqish JG, Galustians J, Schuller R (2008). Effect of a dental water jet with orthodontic tip on plaque and bleeding in adolescent patients with fixed orthodontic appliances. Am. J. Orthod. Dentofacial Orthop..

[17] Goyal CR, Lyle DM, Qaqish JG, Schuller R (2013). Evaluation of the plaque removal efficacy of a water flosser compared to string floss in adults after a single use. J. Clin. Dent..

[18] van der Maarel-Wierink CD, Vanobbergen JN, Bronkhorst EM, Schols JM, de Baat C (2013). Oral Health care and aspiration pneumonia in frail older people: A systematic literature review. Gerodontology.

[19] Müller F (2015). Oral hygiene reduces the mortality from aspiration pneumonia in frail elders. J. Dent. Res..

[20] Quince C, Walker AW, Simpson JT, Loman NJ, Segata N (2017). Shotgun metagenomics, from sampling to analysis. Nat. Biotechnol..

[21] Torkko H, Asikainen S (1993). Occurrence of Porphyromonas gingivalis with Prevotella intermedia in periodontal samples. FEMS Immunol. Med. Microbiol..

[22] Idate U, Bhat K, Kotrashetti V, Kugaji M, Kumbar V (2020). Molecular identification of Capnocytophaga species from the oral cavity of patients with chronic periodontitis and healthy individuals. J. Oral Maxillofac. Pathol..

[23] Benamghar L, Penaud J, Kaminsky P, Abt F, Martin J (1982). Comparison of gingival index and sulcus bleeding index as indicators of periodontal status. Bull. World Health Organ..

[24] Etiology, W. H. O. S. G. O. E., Prevention of Periodontal, D. & World Health, O. *World Health Organization technical report series ; no. 621* (World Health Organization, Geneva, 1978).

[25] Mühlemann HR, Son S (1971). Gingival sulcus bleeding—A leading symptom in initial gingivitis. Helv. Odontol. Acta.

[26] Lindhe J, Haffajee AD, Socransky SS (1983). Progression of periodontal disease in adult subjects in the absence of periodontal therapy. J. Clin. Periodontol..

[27] Greenstein G, Caton J, Polson AM (1981). Histologic characteristics associated with bleeding after probing and visual signs of inflammation. J. Periodontol..

[28] Loe H, Theilade E, Jensen SB (1965). Experimental gingivitis in man. J. Periodontol..

[29] Valm AM (2019). The structure of dental plaque microbial communities in the transition from health to dental caries and periodontal disease. J. Mol. Biol..

[30] Marsh PD (2004). Dental plaque as a microbial biofilm. Caries Res..

[31] Baker JL (2021). Deep metagenomics examines the oral microbiome during dental caries, revealing novel taxa and co-occurrences with host molecules. Genome Res..

[32] Loe H, Silness J (1963). Periodontal disease in pregnancy. I. Prevalence and severity. Acta Odontol. Scand..

[33] Page RC, Schroeder HE (1976). Pathogenesis of inflammatory periodontal disease. A summary of current work. Lab. Invest..

[34] Turesky S, Gilmore ND, Glickman I (1970). Reduced plaque formation by the chloromethyl analogue of victamine C. J. Periodontol..

[35] Callahan BJ (2016). DADA2: High-resolution sample inference from Illumina amplicon data. Nat. Methods.

[36] Caporaso JG (2010). QIIME allows analysis of high-throughput community sequencing data. Nat. Methods.

[37] Segata N (2011). Metagenomic biomarker discovery and explanation. Genome Biol..

[38] Beghini F (2021). Integrating taxonomic, functional, and strain-level profiling of diverse microbial communities with bioBakery 3. Elife.

[39] Bolger AM, Lohse M, Usadel B (2014). Trimmomatic: A flexible trimmer for Illumina sequence data. Bioinformatics.

[40] Langmead B, Salzberg SL (2012). Fast gapped-read alignment with Bowtie 2. Nat. Methods.

[41] Buchfink B, Xie C, Huson DH (2015). Fast and sensitive protein alignment using DIAMOND. Nat. Methods.

