# Supplementary figures and images for: Effect of a multichannel oral irrigator on periodontal health and the oral microbiome
Source: Sci Rep. 2023 Jul 25;13:12043. doi: 10.1038/s41598-023-38894-0 (PMC10368725; doi:10.1038/s41598-023-38894-0)

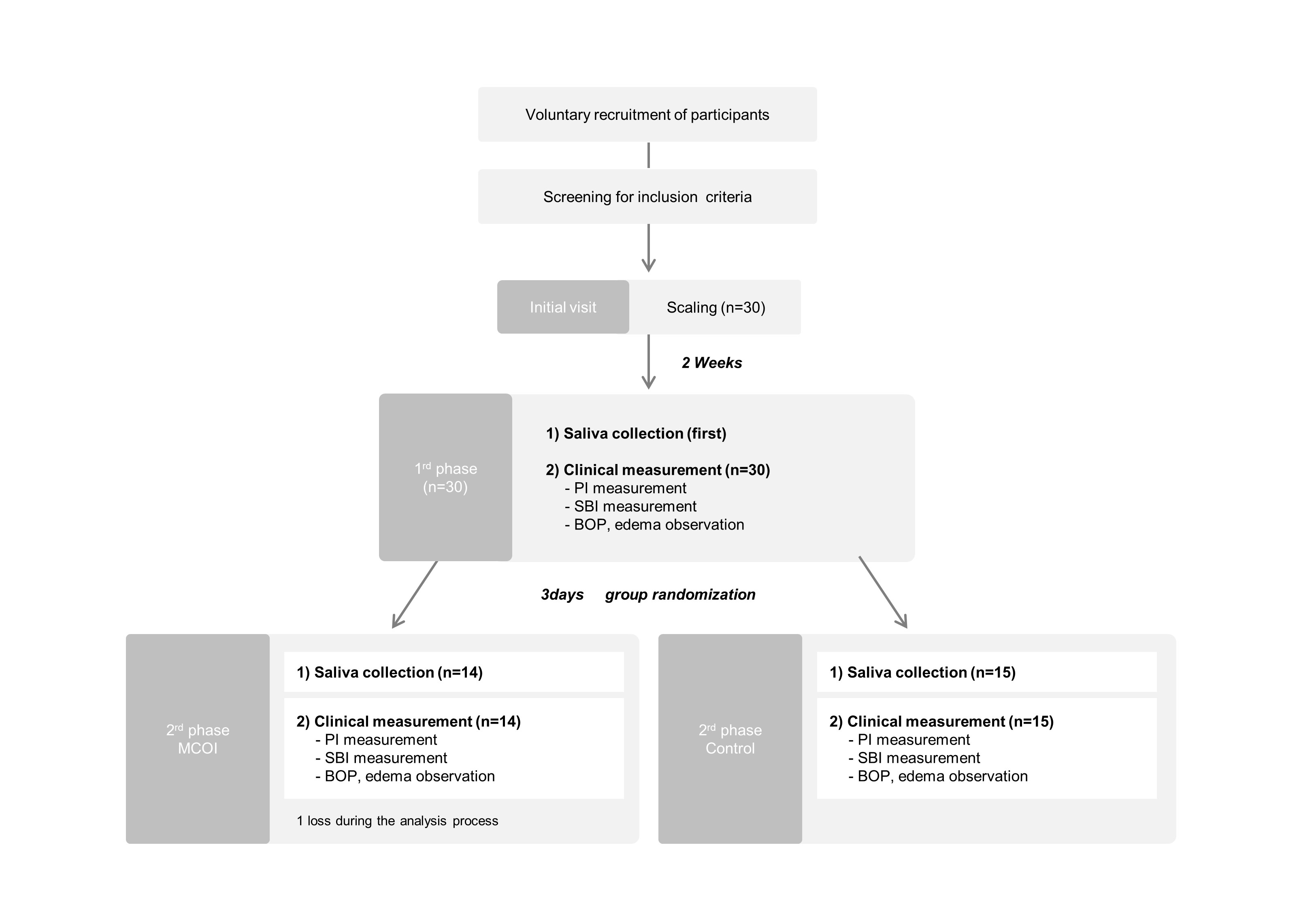

Supplement: Supplementary file 1 — Supplementary Figure S1. [file 41598_2023_38894_MOESM1_ESM.jpg]

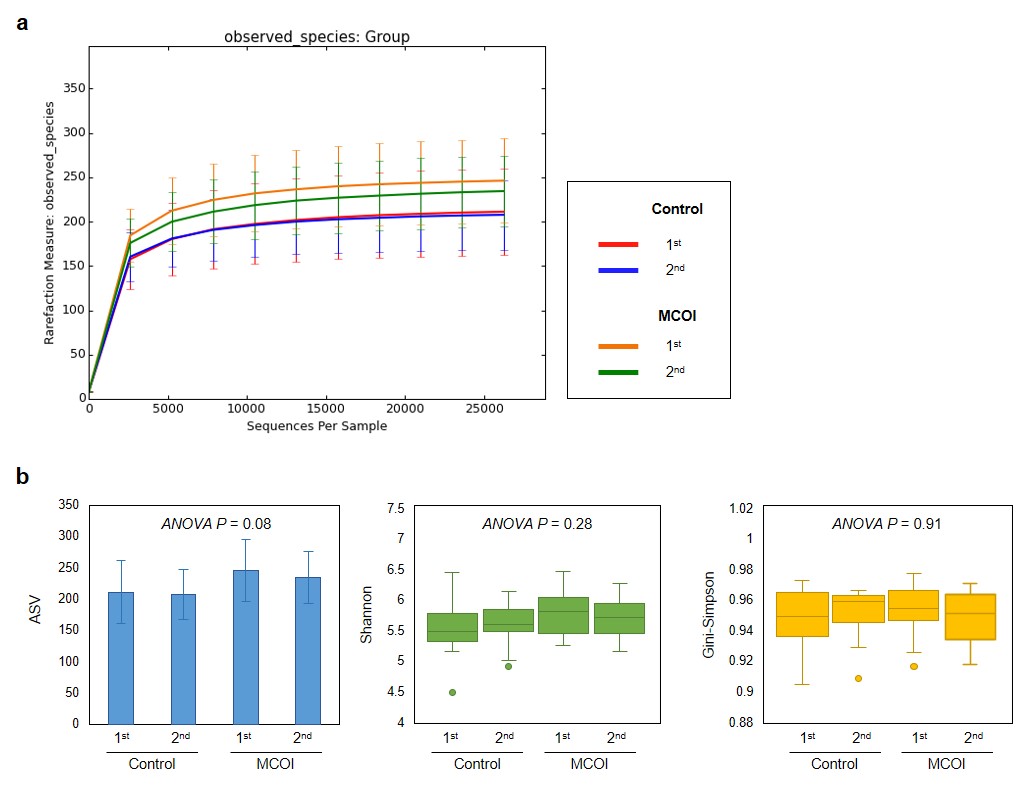

Supplement: Supplementary file 2 — Supplementary Figure S2. [file 41598_2023_38894_MOESM2_ESM.jpg]

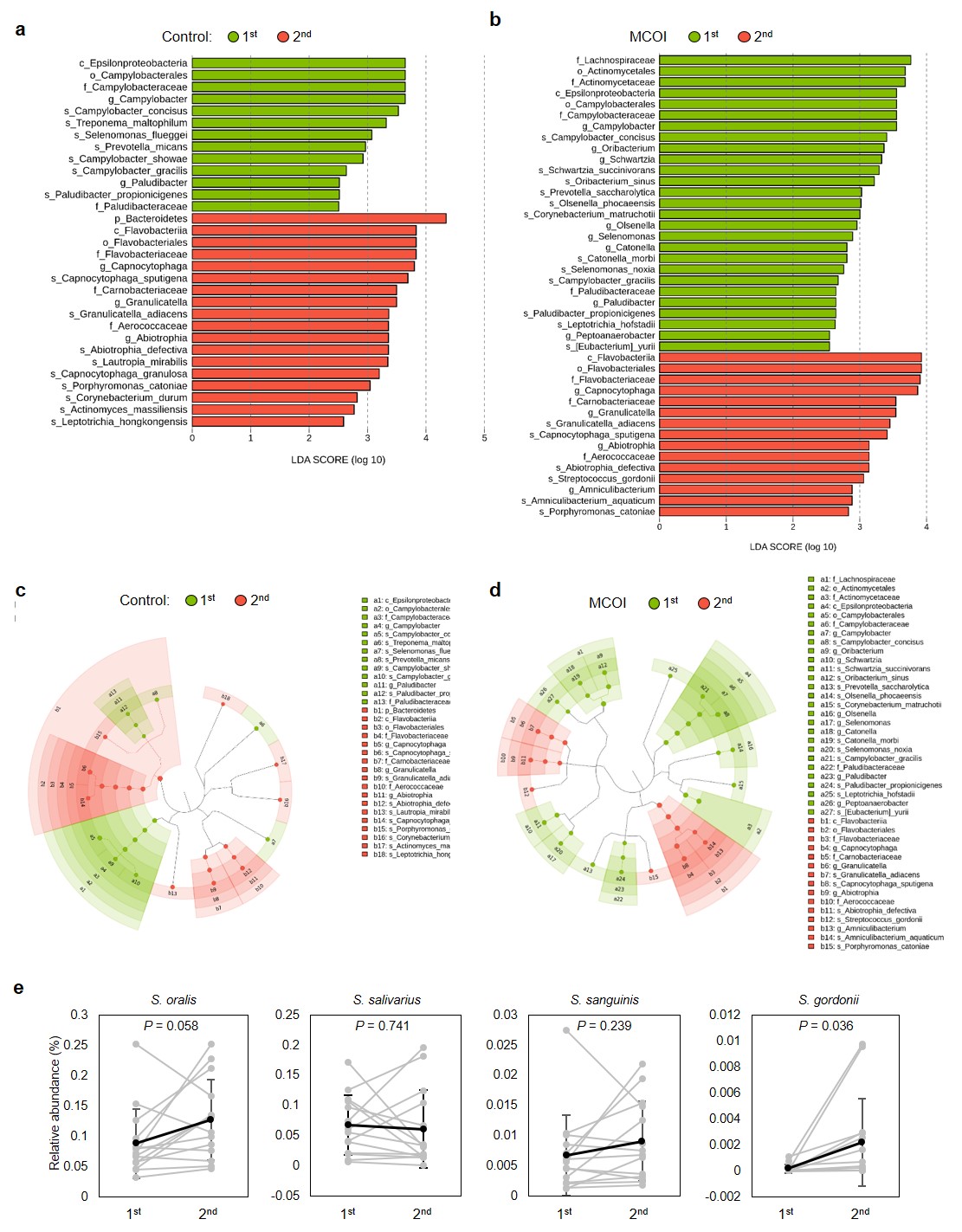

Supplement: Supplementary file 3 — Supplementary Figure S3. [file 41598_2023_38894_MOESM3_ESM.jpg]
