# Supplementary material for: Effect of a multichannel oral irrigator on periodontal health and the oral microbiome
Source: Sci Rep. 2023 Jul 25;13:12043. doi: 10.1038/s41598-023-38894-0 (PMC10368725; doi:10.1038/s41598-023-38894-0)
Supplement: Supplementary file 4 — Supplementary Figure Legends. [file 41598_2023_38894_MOESM4_ESM.pdf]

Supplementary Figure legends

Supplementary Figure S1. Flowchart of the clinical trial. Twenty-nine of 30 participants were finally included for clinical measurements; 1 participant was excluded because of loss during the analysis process. Saliva collection and clinical measurement were performed in all 29 participants. BOP, bleeding on probing; PI, Quigley Hein Plaque Index; SBI, Mühlemann-Son Sulcus Bleeding Index.

Supplementary Figure S2. Results of alpha diversity in the control and multichannel oral irrigator (MCOI) groups. (a) Rarefaction curves of the samples. Data are presented as mean ± standard error of the mean. (b) Alpha diversity results of amplicon sequence variants (ASV), Shannon index, and Gini-Simpson index. N = 15 (control) and 14 (MCOI). ANOVA, analysis of variance.

Supplementary Figure S3. Linear discriminant analysis effect size (LEfSe) analysis of the control and multichannel oral irrigator (MCOI) groups. (a and b) Histograms of linear discriminant analysis scores of differential species in the first and second phases in the control (a) and MCOI (b) groups. (c and d) Cladograms of LEfSe of differential species in the first and second phases in the control (c) and MCOI (d) groups. (e) Relative abundance of four selected *Streptococcus* spp. in the MCOI group in the first and second phases. Black and gray lines indicate values of the average and individual sample, respectively. n = 14; data are presented as mean ± standard error of the mean.
